# Primary thymic mucosa-associated lymphoid tissue lymphoma complicated with renal amyloidosis: A first case report

**DOI:** 10.1097/MD.0000000000019462

**Published:** 2020-03-27

**Authors:** Qin Chen, Yongjing Du, Singh Prince, Ping Zhang, Guisen Li, Li Wang, Wei Wang

**Affiliations:** a Department of Nephrology and Institute of Nephrology, Sichuan Academy of Medical Science and Sichuan Provincial People's Hospital, Chengdu, China; b Division of Nephrology and Hypertension, Mayo Clinic, Rochester, Minnesota, USA.

**Keywords:** MALT lymphoma, monoclonal immunoglobulinemia, renal amyloidosis, thymus

## Abstract

**Introduction:**

Primary mucosa-associated lymphoid tissue (MALT) lymphomas originating in thymus is rare. And, there have been few reports of patients with MALT coexisting with amyloidosis. As far as we know, this was the first case report on MALT lymphoma associated with renal amyloidosis.

**Patient concerns:**

A 57-year-old man presented with nephrotic syndrome. Further workup revealed IgM-Lambda type monoclonal gammopathy. Bone marrow biopsy showed 8% clonal plasma cells. Renal biopsy confirmed the diagnosis of Lambda light chain AL amyloidosis. positron emission tomography/computed tomography showed thymic lesions which upon biopsy were diagnosed as MALT lymphoma of the thymus.

**Diagnosis:**

Primary thymic MALT lymphoma complicated with renal amyloidosis.

**Interventions:**

The patient underwent surgical resection of the thymus mass and 2 courses of chemotherapy.

**Outcomes:**

Follow-up data showed that the patient survived 18 months after surgical excision and chemotherapy.

**Conclusion:**

The case highlights the importance of screening for malignancy in patients with renal amyloidosis.

## 1 Introduction

Mucosa-associated lymphoid tissue (MALT) lymphoma is an extranodal variant of marginal zone B-cell lymphomas that usually occurs in the gastrointestinal tract, parotid gland, lungs, accounting for approximately 7% to 8% of non-Hodgkin's malignant lymphoma (NHL).^[1,2]^ MALT lymphoma originating within thymus is rare. In our case, thymic MALT lymphoma with monoclonal immunoglobulinemia and renal amyloidosis was reported. We used “thymus,” “mucosa-associated lymphoid tissue lymphoma” combined with “renal amyloidosis” as a search term on Pubmed. We could not find specific literature on this topic. To our knowledge, this was the first case report on MALT lymphoma complicated with renal amyloidosis. Furthermore, we reviewed the relevant literatures.

## 2 Case report

### 2.1 Clinical history and laboratory findings

A 57-year-old-man presented to Sichuan Provincial People's Hospital in September, 2016 due to bilateral leg edema. He had a history of hepatitis B for eight years prior to his current presentation of edema. Vitals revealed hypotension (89/69 mm Hg) and physical examination was impressive for edema in legs. Laboratory examination after admission was shown in Table 1. He underwent bone marrow biopsy which showed 8% clonal plasma cells. Repeat bone marrow biopsy showed increasing clonal cells but less than 10%. The screening of flow cytology (cluster of differentiation [CD]2, CD7, CD10, CD13, CD15, CD19, CD20, CD33, CD34, CD45,CD117, HLA-DR) revealed no obvious abnormal immunophenotypic cells. The genome analysis of L365P and MYD88 was negative.

**Table 1:**
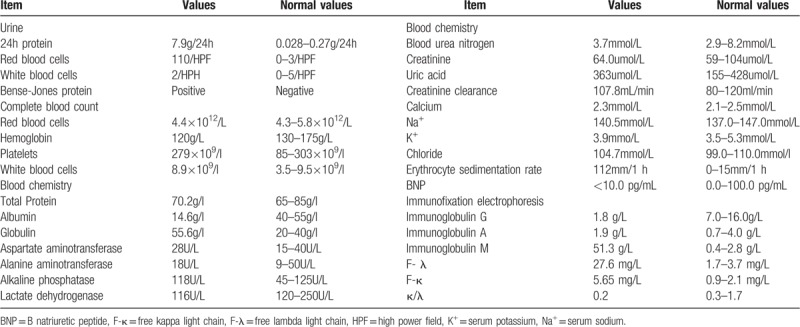
Laboratory data on admission.

### 2.2 Renal biopsy findings

**Figure 1:**
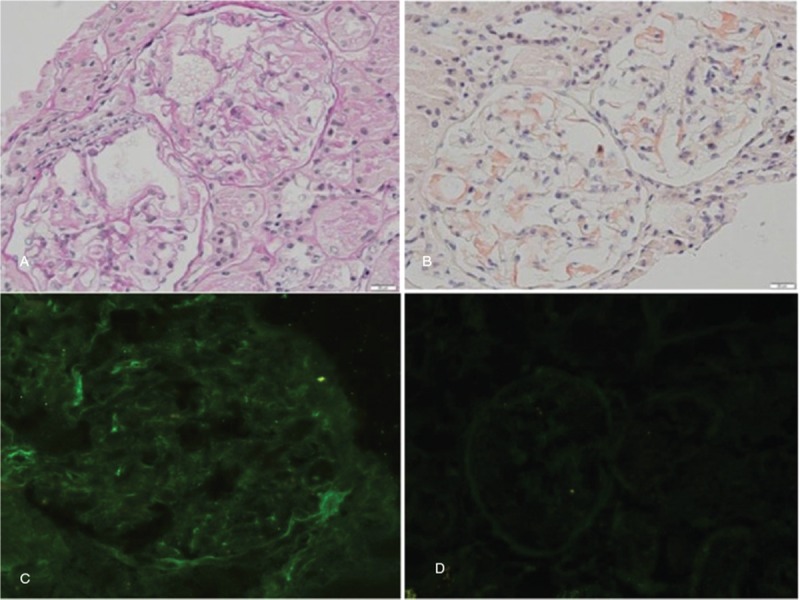
Renal biopsy findings consistent with IgM-λ type amyloidosis. A. Periodic acid Schiff reaction (PAS) staining. In the mesangial area, massive homogeneous and unstructured material deposits were observed (PAS, x400). B. Congo red staining. Renal mesangial area, segmental capillary wall positive (Congo red staining, X400) C. Immunofluorescence lambda light-chain, segmental mesangial block deposition (IF, X400) D. Immunofluorescence lambda light-chain, negative for kappa light-chain (IF, X400).

**Figure 2:**
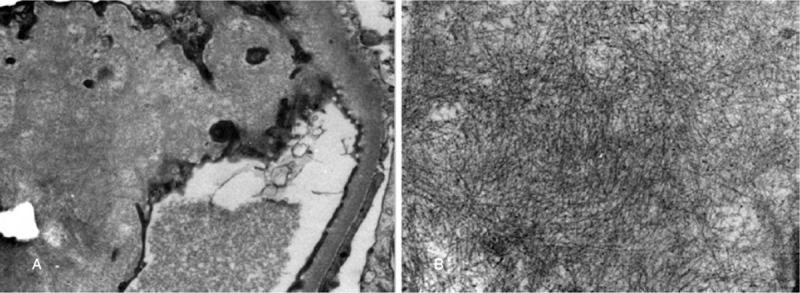
Electron micrograph shows. A. Fibrillary deposits in the mesangial (×12,000). B. Randomly oriented fibrils measuring 8 to 10 nm in diameter (×40,000).

On light microscopy of the renal tissue (2 pieces), 8 glomeruli were seen. Most of the mesangial areas showed massive homogeneous and unstructured material deposition with segmental basement membrane thickening (Fig. 1 A). Congo red staining was positive in glomerular mesangial area along with segmental capillary wall (Fig. 1 B). Immunofluorescence revealed block deposition of Lambda in segmental mesangial area (Fig. 1C). Electron microscopy showed randomly oriented fibrils of 8 to 10 nm in diameter in the mesangium (Fig. 2 B). The pathological diagnosis was AL type amyloidosis.

### 2.3 Positron emission tomography/computed tomography (PET/CT) and thymus biopsy findings

**Figure 3:**
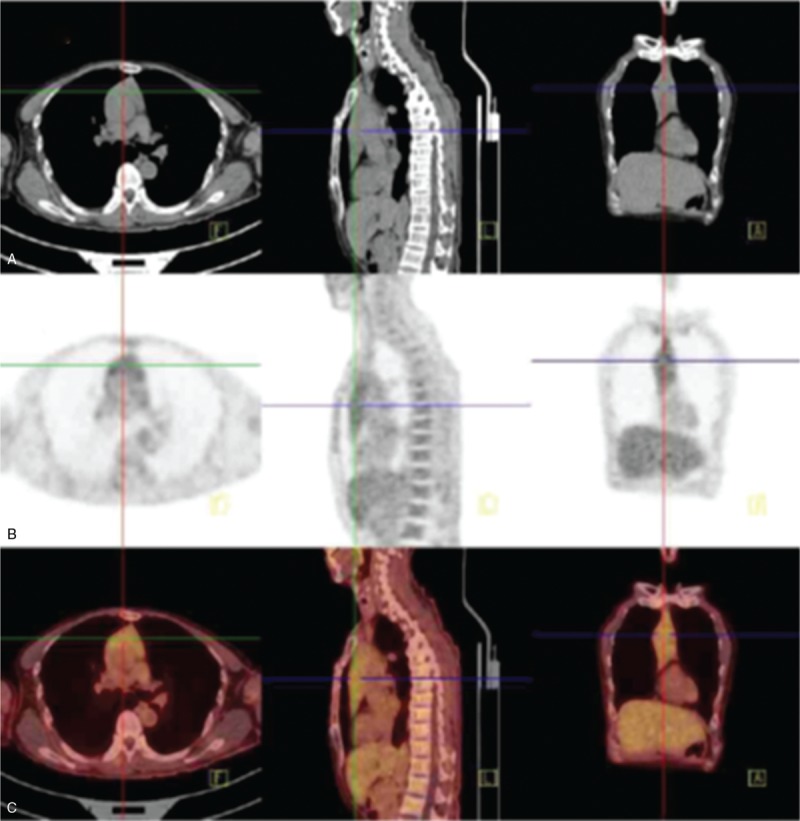
Positron emission tomography-CT (PET-CT) scan showing increased fluorodeoxyglucose uptake in an anterior mediastinal mass with relation to the thymus. CT (A), PET (B) and PET/CT-fused (C) images confirming the focal 18F-FDG uptake. 18F-FDG = 18F-fluorodeoxyglucose.

18F-fluorodeoxyglucose PET/CT was performed in patients after fasting state. PET/CT showed soft tissue density in the anterior mediastinum and thymus region, with slightly active fluorodeoxyglucose metabolization (Fig. 3). The maximum standardized uptake value was 2.7 and the maximum diameter of the lesion was 5.1x4.0x11.3 cm. Though the lesions likely represented thymoma, biopsy of thymus was considered given relatively higher pre-test probability of malignancy. Immunohistochemical staining revealed CD20(+), CD3 scattered (+), TdT(-), CD99(-), CD5(-), CD23 (-), CyclinD1 (-), TIF-1 (-), ki67 positive for 2%, CK staining showed residual thymic epithelial cells were positive. The results of pathological examination were consistent with thymic mucosa-associated lymphoma. A final diagnosis of thymic MALT lymphoma associated with renal amyloidosis was made.

### 2.4 Clinical follow-up

The patient underwent surgical resection of the thymus mass and 2 courses of chemotherapy with CHOP (cyclophosphamide + doxorubicin + vincristine + prednisone) Following the course of treatments, the patient's serum albumin increased to 2.2.1 mg / dL, while 24 h urine protein decreased to 3.78 g / 24 h. Free Lambda chain decreased to 12.6 mg/L. This patient refused further treatments because severe lung infection during the second chemotherapy. In the end, the patient was lost to follow-up.

The patients signed informed consents to publish of this case report and relevant images.

## 3 Discussion

Primary thymic extranodal MALT is a rare lymphoma. In the East Asian population, it is seen in women around 50 years of age. The disease usually follows an indolent course.^[3]^ We have presented an exceedingly rare case of MALT lymphoma of the thymus complicated with renal amyloidosis found in our hospital. Based on the initial clinical presentation together with laboratory findings and renal biopsy, a diagnosis of light Lambda chain Immunoglobulin Light chain amyloidosis (AL) amyloidosis was proposed, but after a mediastinal mass was detected on PET-CT scan, a thymic neoplasm was suspected and a subsequent thymus gland biopsy showed lots of lymphocytes, with immunohistochemical staining positive for CD20 with scattered CD3 positivity. ki67 positive rate was 2% as well as CK staining showed residual thymic epithelial cells to be positive as well.. Finally, the patient was diagnosed with thymus primary MALT lymphoma complicated with renal amyloidosis. To our knowledge, this was the first case report on MALT lymphoma complicated with renal amyloidosis.

Through literature review, we found that monoclonal gammopathy has been reported in many lymphoproliferative diseases. For example, patients with B-chronic lymphocytic leukemia, Burkitt lymphoma, well-differentiated lymphoma, and other subtypes of lymphomas have been described. In patients with indolent B lymphoma, 20% have M proteinemia.^[1,2]^ The literature reported 7107 patients with monoclonal immunoglobulinemia, and 377 (5.3%) were IgM-type monoclonal immunoglobulinemia. Of the 377 patients, 157 (41.6%) were undefined monoclonal immunoglobulinemia (MGUS) and 105 (27.9%) were Waldenstrom's macroglobulinemia. 69 cases (18.3%) were non-Hodgkin B-cell lymphoma, and the rest were primary amyloidosis, cryoglobulinemia and other diseases.^[4–6]^ Cao et al ^[7]^ reported 255 patients with B-cell NHL and 44 patients (17.25%) with M proteinemia. There were 22 cases(8.6%) with IgG type, 4 cases(1.6%) with IgA type and 18 cases(7.0%) with IgM type. Of the 255 NHL patients, 166 were aggressive NHL lymphomas, mainly consisted of diffuse large B-cell lymphomas and follicular lymphomas; 89 were indolent lymphomas, MALT lymphomas and chronic Lymphocytic leukemia were most common. Pangalis et al ^[8]^ analyzed the etiology of 130 patients with IgM monoclonal immunoglobulinemia, 84 patients were lymphoplasmacytic lymphoma, while 5 patients had unexplained IgM monoclonal immunoglobulin. 41 patients diagnosed with other B-cell disorders including 9 cases of B-cell chronic lymphocytic leukemia, 14 cases of marginal zone lymphoma, and small lymphocytic lymphoma, follicular lymphoma, mantle cell lymphoma, cryoglobulinemia and so on. From all above we can see that secreting monoclonal immunoglobulin is not a unique feature of plasma cell disease, and many other B cell tumors are also associated with M proteinemia.^[9]^

Economopoulos et al found that MGUS in B-cell NHL was associated with advanced stage and had a negative prognostic factor in aggressive B-cell NHL.^[4]^ Kyle et al reported 430 patients with monoclonal IgM serum protein and found that more than two-thirds of patients died after more than 20 years of follow-up. The most common cause of death was a malignant lesion of lymphoma.^[5]^ 213 patients with IgM MGUS were diagnosed in Minnesota from 1960 to 1994. The patients were followed up for a total of 1567 person-years. 17 patients developed lymphoma and 6 developed to Waldenstrom's macroglobulinemia, 3 developed primary amyloidosis.^[6]^

Next, we reviewed the literature to understand the renal pathological changes associated with IgM monoclonal gammopathy. Data from Mayo Clinic showed that of the 1363 patients with B-cell lymphoproliferative or Waldenström macroglobulinemia there were 57 who underwent renal biopsy. Two out of 57 patients were diagnosed as marginal zone lymphoma. The most common monoclonal gammopathy–related kidney lesion was monoclonal Ig–related amyloidosis, followed by cryoglobulinemic GN.^[10]^ In our patient, we hypothesized that thymic MALT lymphoma may secrete IgM- Lambda M protein. Furthermore, we searched the literature to understand the clinical features of MALT lymphoma with M proteinemia.

The Chinese literature reported the clinical features of 16 patients with MALT lymphoma, 6 of 16 patients with M proteinemia, and 4 of 6 patients were IgM type (3 cases of Kappa light chain, 1 case of Lambda light chain), 2 cases were IgG type (both K light chain).^[11]^ It is known that MALT lymphoma possesses unique clinicopathological features. Previous studies suggest that a higher incidence of M-proteinemia may be associated with a higher number of monoclonal plasma cells.^[12]^ This case was consistent with the previous research, as there was 8% monoclonal plasma cells in the bone marrow.

MALT lymphoma often contains a large number of monoclonal plasma cells, and it is speculated that MALT cells may be derived from B-cells in the border area of the postgerminal center. Such cells predate plasma cells during B-cell development and have completed rearrangement and mutation of immunoglobulin heavy and light chain genes.^[13]^ However, the marginal zone also includes a highly heterogeneous group of precursor B-cells that can differentiate into plasma cells and secrete low affinity antibodies. MALT lymphoma usually contains a large number of monoclonal plasma cells, and according to the report as many as 30% of original diagnosed MALT lymphoma patients can be accompanied by monoclonal plasma cells.^[14,15]^

It is worth mentioning that IgM type is the least common in patients with different types of monoclonal immunoglobulinemia, and thymus primary MALT lymphoma with IgM type monoclonal immunoglobulinemia is seen more rarely. We reported this patient with nephrotic syndrome as the first manifestation, combined with type IgM- Lambda monoclonal immunoglobulinemia, and renal biopsy suggesting AL amyloidosis, and thymic biopsy suggesting thymic MALT lymphoma.

The Chinese literature reported a case of stomach and duodenal MALT lymphoma complicated with gastrointestinal amyloidosis, suggesting that this type of lymphoma can cause gastrointestinal amyloid, easily misdiagnosed as duodenal ulcer and other diseases.^[16]^ Previously literature reports two cases of thyroid MALT lymphoma with associated amyloid protein deposition.^[17]^ With regard to our patient, we hypothesized that the secretion of IgM-Lambda monoclonal immunoglobulin in the primary thymus MALT lymphoma may lead to renal amyloidosis in the patient.

In summary, we report for the first time a case of thymus gland MALT lymphoma with IgM-Lambda type renal amyloidosis. It would thus be helpful for nephrologists to entertain this diagnosis as a possibility when evaluating patients with renal amyloidosis disease.

## Author contributions

All authors have read and approved the manuscript for submission. Data collection (Qin Chen, Yongjing Du, Ping Zhang, Wei Wang), study design (Wei Wang, Guisen Li, Li Wang), writing (Yongjing Du, Wei Wang), language modification (Singh, Prince).

**Data curation:** Qin Chen, Yongjing Du, Ping Zhang.

**Methodology:** Qin Chen, Guisen Li, wei wang.

**Project administration:** Guisen Li, Li Wang.

**Resources:** Ping Zhang, wei wang.

**Supervision:** Li Wang.

**Writing – original draft:** Yongjing Du.

**Writing – review & editing:** Singh Prince, wei wang.
